# Heart Failure Awareness Among the General Saudi Population: A Cross-Sectional Study

**DOI:** 10.7759/cureus.42077

**Published:** 2023-07-18

**Authors:** Mohammad R Alshammri, Fadhah Saud Alhudayris, Lama Khalid Alshuaibi, Bassam Abdulaziz Alhusaini, Omar Abdulaziz Alfozan, Abdulmalak Abdullah Alsaleh, Saad A Alzmamy

**Affiliations:** 1 Department of Cardiology, Imam Mohammad Ibn Saud Islamic University, Riyadh, SAU; 2 Department of Medicine, Imam Mohammad Ibn Saud Islamic University, Riyadh, SAU; 3 Department of Orthopedic Surgery, Imam Mohammad Ibn Saud Islamic University, Riyadh, SAU

**Keywords:** knowledge, public, saudi arabia, awareness, heart failure

## Abstract

Background

Heart failure (HF) continues to be a globally prevalent condition with a poor prognosis, severe morbidity, and a high mortality rate. Despite the severity of HF, relatively few studies on public awareness of the condition have been published, with the majority indicating that awareness is quite low. This study aimed to determine HF knowledge in the general Saudi community and its associated predictors.

Methods

An online survey was used to conduct a cross-sectional study on the Saudi population. The publicity committee of the Korean Society of Heart Failure drafted the questionnaire used in the present investigation. Three questions assessed knowledge of cardiovascular (CV) and cerebrovascular disorders; four questions assessed knowledge of HF, its etiology, and severity; and three questions assessed knowledge of readmission, mortality, and lifetime risk.

Results

A total of 1,124 respondents completed the questionnaire. Approximately half of the respondents (50.1%, n = 563) were unaware that HF is a pathological rather than a physiological process. Only 13.8% of the respondents were aware that the lifetime risk of developing HF is 20%, with even lower rates of correct responses for the one-year readmission rate (7.4%) and post-discharge one-year mortality from acute HF (7.3%). Female gender and lower levels of education were associated with a lack of HF awareness. A multivariate analysis revealed that income and information source were substantially associated with cardiovascular disorder knowledge. Age, education, alcohol consumption, and information source were associated with awareness of the severity of HF.

Conclusion

The general population in Saudi Arabia (SA) exhibited a relatively low degree of knowledge of HF. We suggest increasing public awareness of HF through an educational campaign led by medical personnel and disseminated via various social media websites. Changes should be made to national healthcare policies to provide healthcare institutions with continuous promotion and iterative campaigns about healthy lifestyles and preventive activities to reduce disease-related costs and disability. HF awareness must be raised through increased concentration and education.

## Introduction

Heart failure (HF) continues to be a worldwide epidemic with a poor prognosis, high morbidity, and high mortality rate [1]. It affects 26 million people worldwide and contributes to the global rise in healthcare costs. Likewise, the medical expenses associated with treating HF patients are substantial, which places an enormous financial burden on the global population [2,3]. Despite recent advances in medications and technology that help people with HF live longer [4,5], the prognosis for HF remains bleak, even worse than for cancer [6-8]. Consequently, additional therapies are always necessary to improve the prognosis of HF. To improve the treatment and prevention of HF, medical personnel and the general public must first be informed about the condition. Despite the severity of HF, only a handful of studies on HF knowledge in the general population have been published, with the majority indicating that public awareness of the condition is rather limited [9].

Despite recent remarkable advances in HF treatment [10], including the discovery of disease mechanisms, characteristics such as educational or socioeconomic factors and their relationship with knowledge and awareness of HF have been relatively understudied and are of little interest in the majority of studies. In this regard, public awareness of HF is an important aspect of disease burden management. Correct identification of HF symptoms, severity, and prognosis permits early access to medical systems and accurate diagnosis and treatment.

Since 1990, there has been minimal research on general population HF knowledge, primarily in Europe [11-14]. In accordance with these findings, the identification rate of HF in the general population was quite low. In the 2005 Study of Heart failure Awareness and Perception in Europe (SHAPE) study conducted in nine European countries, it was determined that the general public's knowledge of HF is very limited [13]. In spite of the fact that 86% of respondents had heard of heart failure, this study indicated that just 3% of them could accurately identify the condition from a list of typical symptoms and indicators, while 31% properly identified angina and 51% correctly identified transient ischemic attack/stroke. Only 29% of respondents believed that HF symptoms and indicators indicate a "severe" illness [13]. Only 3% of individuals understood the symptoms of heart failure, compared to 31% for angina and 55% for ischemic stroke. Similarly, European research conducted in the 2010s revealed little progress in HF awareness [11,14].

In Asia, the prevalence of HF continues to rise, resulting in substantial healthcare costs. However, few studies were conducted to examine the general population's knowledge and cognizance of HF. A study conducted in Korea revealed that public awareness of HF remains low [15]. To be more specific, although 80% of participants had heard of HF, only 47% could precisely describe it. A minority of participants correctly identified the lifetime risk of developing HF (21%), as well as its associated mortality (16%), readmission risk (18%), and economic burden (28%) [15]. A substantial proportion of participants misidentified HF symptoms and grossly underestimated the risk of HF. Notably, more than half of the participants misunderstood the appropriate physical exercises for HF patients [15]. According to a previous study of the knowledge of the geriatric population, approximately 70% of the participants had an incorrect comprehension of the term "heart failure." In addition, over 90% of the participants in this study expressed a desire for more education and information about heart failure for both patients and their caregivers [16]. According to a study that looked at public awareness of HF in 13 different countries, despite that 99% of the general public respondents had heard of HF, only 6% correctly identified shortness of breath, fatigue, and leg swelling as the main symptoms of HF [17].

Despite the high prevalence of heart failure in the Middle East, the economic impact of chronic heart failure is underrecognized compared to developed nations [18]. It was estimated that 1.35 million patients are being treated for HF in the Middle East, with approximately 320,000 in Saudi Arabia (SA). The estimated cost of the disease, inclusive of treatment and complications, was $1.92 billion. The United Arab Emirates (UAE) had the highest annual costs per patient [19], followed by South Africa and Egypt. Few studies have been conducted in the Middle East to assess knowledge and awareness of heart disease; almost none have focused on HF. Research in the UAE revealed that Emirati women, the intended audience for the questionnaire, lack information about the severity and susceptibility to heart disease in the region, which is crucial for enhancing cardiovascular (CV)-related health outcomes [20]. This study required a greater emphasis on gender- and age-specific knowledge of the risks and symptoms of cardiac disease. The study also identified potentially modifiable barriers to procuring healthcare that should be addressed to reduce heart disease-related morbidity and mortality among UAE native females.

According to the World Heart Federation, 55% of individuals did not recognize a description of HF, and 67% incorrectly identified diabetes, elevated blood pressure, and coronary heart disease as primary risk factors for developing HF [19]. Despite the prevalence of risk factors in the Middle East and North Africa (MENA), the management of hypertension and dyslipidemia is suboptimal. Of participants with poor incomes, 70% had dyslipidemia, but only 4% received lipid-lowering medication [20,21].

These disparities in the severity and perception of HF can ultimately hinder treatment and worsen the prognoses of HF patients [22]. Therefore, it is essential to identify the components of HF awareness to increase it. Controlling risk factors for HF would significantly reduce progression to HF, readmission rates, and premature mortality in HF. Keeping this in mind, the purpose of this study was to ascertain the HF knowledge of the general Saudi population and its associated predictors.

## Materials and methods

Study design and settings

The aim of this online cross-sectional study conducted between October and December 2022 was to assess Saudi populations' knowledge of HF. This research made use of a non-probability convenience sampling technique. The research team collected data using an online questionnaire that was disseminated via social media websites (Instagram, Facebook, and Snapchat).

Sample population

Our research population consisted of all Saudi Arabians over the age of 18 who are currently residing in the country. Participants who did not meet our inclusion criteria were excluded. The survey's cover letter highlighted the inclusion criteria. Those who did not satisfy the inclusion criteria were asked to leave the study. Based on our exclusion criteria, a total of 247 participants were excluded.

Questionnaire instrument

The questionnaire used in this study was devised by the publicity committee of the Korean Society of Heart Failure. By reviewing/revising previous surveys and adding a number of new questions that reflect local factors, the questionnaire items were carefully selected. Age, sex, and residence questions preceded the primary survey to validate the eligibility of the study population. Similar to the questionnaire administered by Remme et al. [13], this questionnaire shared a similar structure. Two sections comprised the primary survey. One section describes the perception of the disease prior to the definition of HF, while the other describes the awareness and knowledge of the disease (etiology, severity, healthcare cost, prognosis, and treatment) after the definition of HF was provided. The HF questionnaire was followed by questions regarding other demographic factors, including education, average income, the presence of disease (in the respondent or their family), smoking, and alcohol consumption.

To assess knowledge of cardiovascular (CV) diseases and HF, some of the included queries were categorized as follows: (1) knowledge of various cardiovascular and cerebrovascular conditions (three questions), (2) knowledge of HF, its etiology, and severity (four questions), and (3) knowledge of readmission, mortality, and longevity risk (three questions). Appendix 1 contains the queries associated with each section. None of the included demographic variables was substantially associated with knowledge of readmission, mortality, and the lifetime risk of developing HF (three questions). Consequently, it was not evaluated separately. Nonetheless, a total score of 10 was assigned to the three domains when calculating the aggregate knowledge score.

Face validity check and piloting phase

The translation of the questionnaire tool followed the forward-backward translation technique. The Arabic translation of the questionnaire was reviewed by expert clinicians for clarity and readability. They affirmed that the questionnaire items are straightforward and in line with the study's aims. Then, a small pilot research was conducted with a group of participants from the general public. The pilot study affirmed that the questionnaire questions are straightforward and simple to complete.

Sample size calculation

Using a confidence interval of 95%, a standard deviation (SD) of 0.5, and a margin of error of 5%, the minimum sample size required was 385 individuals.

Ethical approval

This study was approved by the Institutional Review Board at Imam Mohammad Ibn Saud Islamic University, Riyadh, Saudi Arabia (12RM0062033). As participation in the study was voluntary, the research ethics committee approved the consent waiver.

Statistical analysis

Statistical analysis was performed using R version 3.6.3. Counts and percentages were used to summarize categorical variables, and the mean ± standard deviation was used for continuous variables. The chi-square test of independence was used to assess the difference between categorical variables and the responses to individual questions. An unpaired t-test was used to compare the mean scores for HF awareness, awareness regarding CV diseases, and total awareness scores between groups. Ordinal logistic regression was used to assess factors associated with awareness regarding different heart failure domains. Hypothesis testing was performed at a 5% level of significance.

## Results

Sociodemographic characteristics

A total of 1,124 respondents completed the questionnaire. Males and females made up 40.2% and 59.8% of the population, respectively. Around 74.3% possessed a bachelor's degree or higher. The majority of respondents were between the ages of 18 and 25 (46%) and 26 and 35 (24%). Around 30.4% of the participants were from the southern area. Around one-third (31.2%) of respondents had a personal or family history of cardiovascular disease, and 4.3% had heart failure. Of the respondents, 74.6% reported no comorbidities; 10.1% had hypertension, 7.9% had diabetes, and 7.4% had dyslipidemia. Three-quarters of the respondents were non-smokers, while 4.18% were alcoholics. Heart failure information was obtained from secondary or tertiary care clinics (41.5%), the Internet (38.5%), and primary care clinics (18.1%) (Table 1).

**Table 1:** Sociodemographic characteristics of the included respondents MI: myocardial infarction

Variable	Frequency (%)
Gender	
Female	672 (59.8%)
Male	452 (40.2%)
Age	
18-25 years	517 (46%)
26-35 years	270 (24%)
36-45 years	183 (16.3%)
>45 years	154 (13.7%)
Area of residency	
Eastern area	155 (13.8%)
Western area	252 (22.4%)
Northern area	53 (4.7%)
Southern area	342 (30.4%)
Central area	323 (28.7%)
Education	
Bachelor's degree or higher	835 (74.3%)
High school	248 (22.1%)
Middle school or lower	41 (3.65%)
History/family history of heart disease	
No	773 (68.8%)
Yes	351 (31.2%)
Heart disease	
I do not know	390 (49.1%)
Angina or MI	97 (12.2%)
Heart failure	110 (13.9%)
Valvular heart disease	130 (16.4%)
Abnormality of heart rhythm	67 (8.44%)
Comorbidities	
Diabetes	89 (7.92%)
Dyslipidemia	84 (7.47%)
Hypertension	113 (10.1%)
None	838 (74.6%)
Income (Saudi riyal)	
<4,000	130 (11.6%)
10,000-14,000	227 (20.2%)
15,000-20,000	213 (19%)
5,000-10,000	260 (23.1%)
>20,000	294 (26.2%)
Smoking status	
Current smoker	151 (13.4%)
Ex-smoker	133 (11.8%)
Non-smoker	840 (74.7%)
Alcohol consumption	
No	1,077 (95.8%)
Yes	47 (4.18%)
Presence of heart failure	
None	915 (81.4%)
Family member	161 (14.3%)
Participant	48 (4.27%)
Source of information regarding heart failure	
Internet	430 (38.3%)
Oriental medicine clinic	12 (1.07%)
Pharmacy	13 (1.16%)
Primary care clinic	203 (18.1%)
Secondary or tertiary care clinic	466 (41.5%)

Knowledge and awareness regarding heart failure

Awareness Regarding HF

Results showed that less than one-half of the respondents heard of HF (44.5%, n = 500). Roughly the same number correctly described heart failure (Table 2).

**Table 2:** Awareness regarding heart failure and its severity *Correct answer

Variable	Frequency (%)
Ever heard of heart failure	
No	624 (55.5%)
Yes	500 (44.5%)
Words that best describe heart failure	
Abnormality of heart rhythm	72 (6.41%)
I do not know	163 (14.5%)
Lack of oxygen and blood supply to the heart due to myocardial infarction	223 (19.8%)
The heart cannot pump enough blood around the body*	572 (50.9%)
Weakness of the heart by aging	94 (8.36%)
Perceived severity if you have breathlessness, tiredness, or swollen ankles	
I do not know	134 (11.9%)
Minor illness	153 (13.6%)
Slightly serious illness	462 (41.1%)
Serious illness*	375 (33.4%)
Time to visit the hospital if one has breathlessness, tiredness, or swollen ankles	
Never	34 (3.02%)
One to two days*	712 (63.3%)
Within 1-3 weeks	98 (8.72%)
Within one month	46 (4.09%)
Within one week	234 (20.8%)

Regarding the severity of heart failure, only one-third of the respondents identified HF as a serious illness (33.4%, n = 375), and 11.9% did not know the correct answer. Two-thirds (63.3%) of the respondents answered that they would visit the hospital within "one to two days" if they experienced breathlessness, tiredness, or swollen ankles.

Etiology of HF

Half of the respondents (50.1%, n = 563) did not know that HF is a pathological rather than a physiological process. When the respondents were asked about the factors that precipitate HF, 83.5% (n = 939) chose hypertension, and 16.5% (n = 185) chose lung disease.

Awareness Regarding the Symptoms of Cardiovascular and Cerebrovascular Disorders

Only one-third of the respondents correctly identified angina/myocardial infarction (MI) symptoms (39.5%, n = 444), and a similar number correctly identified stroke symptoms (34.6%, n = 389). Similarly, 32.4% of the respondents knew that breathlessness, tiredness, and swollen ankles are symptoms of HF (32.4%). Only one-third of the respondents correctly knew the symptoms of two or more CV diseases.

Perception of HF-Related Risk

Only 13.8% of the respondents knew that the lifetime risk of developing HF was 20%. Lower rates of correct responses were observed for the one-year readmission rate (7.4%) and post-discharge one-year mortality from acute HF (7.3%). One-third of the respondents did not know the answer to each of the former questions (33.8% and 29.9%, respectively). One-third of the respondents knew the answers to three or more of the four questions related to HF (definition) and its severity (seriousness, referral time, and whether it is a normal aging process).

Our study findings showed good awareness regarding the impact of HF on quality of life (QoL) and five-year mortality. When the participants were asked about "the most likely disease that has the highest mortality within five years after the diagnosis," 30.1% chose myocardial infarction (MI), 38.8% chose HF, 21.9% chose stroke, and 9% chose prostate or breast cancer. More than half of the respondents (58.5%) thought that heart failure had the highest impact on the QoL as opposed to diabetes (20.5%), hypertension (14.4%), and arthritis (6.5%). Responses were variable regarding the average costs per admission from acute HF, with 25.8% of respondents choosing 30,000-50,000 Saudi riyal (SAR). More than two-thirds of the respondents were worried that one of their friends, colleagues, or neighbors who suffers from HF might suddenly die (69.7%, n = 783). The remaining 11.4% (n = 128) and 19% (n = 213) were not worried or did not know, respectively.

Treatment of HF

Regarding the preference for HF treatment outcomes, 64.2% (n = 722) of the respondents wanted a treatment option that could improve the QoL, and 12.7% (n = 143) wanted a treatment option that could make one live longer. The remaining 23% (n = 259) could not decide. When the participants were asked if patients with HF should live quietly and reduce all physical activities, more than half of the participants (55.3%) agreed and 22.5% (n = 253) did not agree. Almost one-half (54.4%) of the respondents agreed that current HF medication could reduce deaths from HF, 63.3% agreed that the current HF medication could improve the quality of life, and only 35.9% agreed that the current HF medications could prevent the occurrence of HF.

When the analysis was stratified by gender, the proportion of males and females who correctly knew the definition of angina, stroke, and HF was not similar across different males and females although some results were statistically significant due to the difference in the distribution of other responses (Table 3). More females knew the correct definition of HF (P = 0.024). More males than females knew that HF is a serious illness (41.4% versus 28%) and that referral to the hospital within 1-2 days is warranted (73% versus 56.8%). More females knew that HF was a normal aging process and that it had the highest impact on the QoL. Overall, the average HF awareness score was significantly higher in males than in females (2.07 ± 1.11 versus 1.91 ± 1.09, P = 0.02). Regarding education (Appendix 1), respondents who had a bachelor's degree had a significantly higher average overall knowledge score than respondents who completed only high school education or less (P = 0.001). The awareness regarding HF was also higher in this group (P < 0.001).

**Table 3:** Gender and awareness regarding CV diseases and HF *Correct answer HF: heart failure, QoL: quality of life, SD: standard deviation, MI: myocardial infarction, GI: gastrointestinal, CV: cardiovascular

Variable	Female	Male	P-value
	N = 672	N = 452	
Chest heaviness that occurs during exertion and disappears with rest			0.011
Angina or MI*	262 (39%)	182 (40.3%)	
GI disorders	41 (6.10%)	16 (3.54%)	
I do not know	92 (13.7%)	81 (17.9%)	
Lung disorders	83 (12.4%)	71 (15.7%)	
Other heart diseases	194 (28.9%)	102 (22.6%)	
Facial paralysis, double vision, and sudden unilateral weakness in the arm			0.691
Angina or MI	143 (21.3%)	91 (20.1%)	
I do not know	118 (17.6%)	94 (20.8%)	
Other heart diseases	59 (8.78%)	37 (8.19%)	
Parkinson's disease, epilepsy, and other brain diseases	120 (17.9%)	73 (16.2%)	
Stroke*	232 (34.5%)	157 (34.7%)	
Breathlessness, tiredness, and swollen ankles			0.013
Angina or MI	56 (8.33%)	58 (12.8%)	
Heart diseases in general	178 (26.5%)	87 (19.2%)	
Heart failure*	215 (32%)	149 (33%)	
I do not know	116 (17.3%)	90 (19.9%)	
Lung disorders	107 (15.9%)	68 (15%)	
Ever heard of heart failure			0.017
No	393 (58.5%)	231 (51.1%)	
Yes	279 (41.5%)	221 (48.9%)	
Words that best describe heart failure			0.024
Abnormality of heart rhythm	32 (4.76%)	40 (8.85%)	
I do not know	89 (13.2%)	74 (16.4%)	
Lack of oxygen and blood supply to the heart due to myocardial infarction	138 (20.5%)	85 (18.8%)	
The heart cannot pump enough blood around the body*	358 (53.3%)	214 (47.3%)	
Weakness of the heart by aging	55 (8.18%)	39 (8.63%)	
Perceived severity if you have the following symptoms: breathlessness, tiredness, or swollen ankles			<0.001
I do not know	76 (11.3%)	58 (12.8%)	
Minor illness	114 (17%)	39 (8.63%)	
Slightly serious illness	294 (43.8%)	168 (37.2%)	
Serious illness*	188 (28%)	187 (41.4%)	
How soon will you go to the hospital if you feel breathlessness, tiredness, or swollen ankles?			<0.001
Never	23 (3.42%)	11 (2.43%)	
One to two days*	382 (56.8%)	330 (73%)	
Within 1-3 weeks	72 (10.7%)	26 (5.75%)	
Within one month	36 (5.36%)	10 (2.21%)	
Within one week	159 (23.7%)	75 (16.6%)	
Agree that heart failure is a normal aging process			0.010
No*	357 (53.1%)	204 (45.1%)	
Yes	315 (46.9%)	248 (54.9%)	
Causes of heart failure			0.402
Hypertension	567 (84.4%)	372 (82.3%)	
Lung disorders	105 (15.6%)	80 (17.7%)	
Correct lifetime risk of developing heart failure			0.002
2 in 100 people	148 (22%)	134 (29.6%)	
5 in 100 people	219 (32.6%)	162 (35.8%)	
10 in 100 people	202 (30.1%)	104 (23%)	
20 in 100 people*	103 (15.3%)	52 (11.5%)	
Highest mortality within five years after diagnosis			0.158
Heart failure	272 (40.5%)	164 (36.3%)	
Myocardial infarction	208 (31%)	130 (28.8%)	
Prostate or breast cancer	57 (8.48%)	47 (10.4%)	
Stroke	135 (20.1%)	111 (24.6%)	
Worried that one of your friends, colleagues, or neighbors who suffers from heart failure might suddenly die			0.087
I do not know	129 (19.2%)	84 (18.6%)	
No	65 (9.67%)	63 (13.9%)	
Yes	478 (71.1%)	305 (67.5%)	
Post-discharge one-year mortality from acute heart failure			0.196
2 in 100 people	161 (24%)	108 (23.9%)	
5 in 100 people	142 (21.1%)	110 (24.3%)	
10 in 100 people	105 (15.6%)	80 (17.7%)	
20 in 100 people*	46 (6.85%)	36 (7.96%)	
I do not know	218 (32.4%)	118 (26.1%)	
Readmission rate within one year after discharge from heart failure			0.447
2 in 100 people	128 (19%)	88 (19.5%)	
5 in 100 people	146 (21.7%)	118 (26.1%)	
10 in 100 people	111 (16.5%)	70 (15.5%)	
20 in 100 people*	49 (7.29%)	34 (7.52%)	
I do not know	238 (35.4%)	142 (31.4%)	
Average healthcare costs per admission from acute heart failure			0.160
<10,000	114 (17%)	75 (16.6%)	
>80,000	116 (17.3%)	90 (19.9%)	
10,000-20,000	144 (21.4%)	117 (25.9%)	
30,000-50,000	182 (27.1%)	108 (23.9%)	
60,000-80,000	116 (17.3%)	62 (13.7%)	
Disease with the greatest impact on the quality of life			0.001
Arthritis	45 (6.70%)	28 (6.19%)	
Diabetes	111 (16.5%)	120 (26.5%)	
Heart failure	419 (62.4%)	239 (52.9%)	
Hypertension	97 (14.4%)	65 (14.4%)	
Preferred treatments if you were a heart failure patient			<0.001
I can not decide	146 (21.7%)	113 (25%)	
Treatment that could improve the quality of life	460 (68.5%)	262 (58%)	
Treatment that prolongs survival	66 (9.82%)	77 (17%)	
Current heart failure medications could reduce death from HF			0.013
No	55 (8.18%)	54 (11.9%)	
I do not know	261 (38.8%)	142 (31.4%)	
Yes	356 (53%)	256 (56.6%)	
Current heart failure medications could improve the QoL in patients with HF			0.592
No	41 (6.10%)	32 (7.08%)	
I do not know	210 (31.2%)	130 (28.8%)	
Yes	421 (62.6%)	290 (64.2%)	
Current heart failure medications could prevent the occurrence of heart failure			0.055
No	178 (26.5%)	102 (22.6%)	
I do not know	271 (40.3%)	169 (37.4%)	
Yes	223 (33.2%)	181 (40%)	
Heart failure patients should live quietly and reduce all physical activity			0.349
No	142 (21.1%)	111 (24.6%)	
I do not know	155 (23.1%)	94 (20.8%)	
Yes	375 (55.8%)	247 (54.6%)	
Source of information regarding heart failure			0.001
Internet	290 (43.2%)	140 (31%)	
Oriental medicine clinic	7 (1.04%)	5 (1.11%)	
Pharmacy	6 (0.89%)	7 (1.55%)	
Primary care clinic	108 (16.1%)	95 (21%)	
Secondary or tertiary care clinic	261 (38.8%)	205 (45.4%)	
Awareness regarding CV diseases (mean (SD))	1.06 (0.97)	1.08 (0.96)	0.676
Awareness regarding heart failure (mean (SD))	1.91 (1.09)	2.07 (1.11)	0.020

Ordinal logistic regression (Table 4) showed that income and source of information were significantly associated with knowledge regarding CV disorders. Higher income (>20,000) was associated with higher odds of scoring a higher number of correct answers than lower income (odds ratio (OR) = 1.53, P < 0.05). Respondents who obtained their information from secondary or tertiary care clinics were more likely to score a higher number of correct answers than respondents who obtained their information from the Internet (OR = 1.44, P < 0.05). Age, education, alcohol consumption, and source of information were associated with awareness regarding heart failure and its severity. Respondents aged 26-35 years (OR = 1.52, P = 0.003) and >45 years (OR = 1.5, P = 0.016) were more likely to score higher in the "knowledge regarding HF" domain than respondents aged <26 years. Respondents who completed only high school were less likely to respond correctly than those with bachelor's degrees (OR = 0.72, P = 0.018). Alcohol consumption was associated with lower knowledge regarding HF symptoms (OR = 0.51, P = 0.019). Respondents who obtained their information from oriental medicine clinics (OR = 0.34, P = 0.043) and pharmacies (OR = 0.29, P = 0.019) were less likely to respond correctly than respondents who obtained their information from the Internet (Table 4).

**Table 4:** Multivariate analysis of factors associated with knowledge regarding heart failure CI: confidence interval, HF: heart failure

	Awareness regarding symptoms of cardiovascular and cerebrovascular disorders			Awareness regarding HF		
Predictors	Odds ratio	95% CI	P-value	Odds ratio	95% CI	P-value
Gender: male versus female	1.07	0.84-1.37	0.569	1.27	0.99-1.62	0.056
Age: <26 years	Reference			Reference		
Age: 26-35 years	0.92	0.69-1.22	0.560	1.52	1.15-2.01	0.003
Age: 36-45 years	0.78	0.57-1.07	0.127	1.05	0.77-1.43	0.781
Age: >45 years	0.91	0.65-1.27	0.577	1.50	1.08-2.09	0.016
Education: bachelor's degree	Reference			Reference		
Education: high school	0.95	0.73-1.24	0.689	0.72	0.55-0.95	0.018
Education: middle school or lower	0.71	0.37-1.35	0.294	0.64	0.35-1.16	0.138
Heart disease: yes versus no	0.82	0.62-1.07	0.143	1.11	0.86-1.44	0.430
Presence of heart failure	Reference			Reference		
Presence of heart failure: family member	1.18	0.82-1.70	0.385	0.82	0.58-1.17	0.276
Presence of heart failure: participant	1.18	0.66-2.09	0.573	1.26	0.74-2.14	0.397
Alcohol consumption: yes versus no	0.81	0.45-1.45	0.479	0.51	0.29-0.89	0.019
Smoking status: non-smoker	Reference			Reference		
Smoking status: current smoker	0.79	0.55-1.12	0.182	0.88	0.62-1.25	0.482
Smoking status: ex-smoker	1.09	0.76-1.54	0.646	1.11	0.78-1.58	0.570
Income: <5,000	Reference			Reference		
Income: 5,000-10,000	0.79	0.54-1.17	0.239	0.71	0.48-1.04	0.082
Income: 10,000-14,000	1.39	0.94-2.07	0.102	0.85	0.57-1.26	0.412
Income: 15,000-20,000	1.39	0.93-2.10	0.110	1.00	0.67-1.49	0.999
Income: >20,000	1.53	1.04-2.25	0.029	0.98	0.67-1.43	0.903
Source of information: Internet						
Source of information: oriental medicine clinic	0.62	0.18-2.13	0.452	0.34	0.12-0.97	0.043
Source of information: pharmacy	0.92	0.30-2.78	0.877	0.29	0.10-0.81	0.019
Source of information: primary care clinic	1.25	0.92-1.71	0.160	0.92	0.68-1.26	0.619
Source of information: secondary or tertiary care clinic	1.44	1.13-1.84	0.003	1.23	0.96-1.56	0.096

## Discussion

The current cross-sectional study investigated the general Saudi population's awareness of HF and other CV diseases. In SA, we demonstrated that all aspects of HF are mainly unknown or ignored by the general population and that several misconceptions exist. In our study, only 50% of the respondents were unaware that HF is a pathological process as opposed to a natural one. The angina/MI symptoms were appropriately identified by just one-third of the responders. The results suggest that the general Saudi population has a low level of awareness regarding heart failure and other cardiovascular diseases. However, HF was perceived to be more dangerous and burdensome than other common chronic conditions such as MI, stroke, and cancer. In the SHAPE study, cancer was viewed as the most pressing issue by the study population [13].

While there are many similarities between HF and cancer, including mortality, there are significant distinctions that must be accounted for in clinical practice and public health interventions [23]. Recent treatment advances have resulted in a substantial improvement in survival for patients with HF [24], whereas the prognosis for cancer patients has not improved as much as for patients with HF [23], despite greater expenditures [25]. In addition, we have more data regarding cancer awareness in the general population, which is generally regarded as adequate and analogous to patient knowledge [26-29].

Additionally, the analysis of the individual queries revealed some intriguing facts. Less than half of respondents were familiar with heart failure, and even fewer had ever heard of it. These figures are lower than those reported in European nations such as Slovenia, where 83% of respondents had heard of HF and 35% believed it was a natural consequence of aging. The latter was also less than the 50% found in this investigation [11]. Despite this, 50% of respondents in the present study correctly defined HF, which is higher than the 30% reported in the Slovenian population, although this could be explained by the 10-year gap between the two studies. A survey of 2,438 individuals in four European countries revealed that 31% believed that HF was a common disease associated with aging, while only 38% were aware of the very poor prognosis following hospitalization for HF [30].

Regarding preference for HF treatment, a distinct difference was observed between males and females, with females favoring options that could ameliorate symptoms and males favoring options that could prolong survival. However, there was a general preference for QoL-improving options, akin to what has been reported in other studies [11]. Research in the UAE revealed that Emirati women, the intended audience for the questionnaire, lack information about the severity and susceptibility to heart disease in the region, which is crucial for enhancing cardiovascular-related health outcomes. This study uncovered the need for a greater emphasis on gender- and age-specific education regarding the risks and symptoms of cardiac disease. The study also identified potentially modifiable barriers to procuring healthcare that should be addressed to reduce heart disease-related morbidity and mortality among UAE native females [31].

The analysis also revealed a significant correlation between education and HF awareness, with 54% of respondents with a bachelor's degree correctly defining HF, compared to 40% of respondents with only a high school diploma. A study in Korea found that female sex and a lower level of education were independently associated with low HF awareness in the general Korean population [9], corroborating the findings of the present study. Azhar et al. [16] found that low HF awareness was associated with male gender, non-Hispanic race, and low educational level; however, the sample size was small (n=182), research participation was limited to the elderly, and no multivariate analysis was performed [30]. The association between female gender and low awareness was supported by the findings of the present study, which revealed that the average awareness score was substantially lower among females than among males. In comparison to females, three-quarters of males reported that referral was necessary within one to two days due to HF symptoms.

The findings of this investigation should be considered in the context of patients' and physicians' perceptions and comprehension of HF. There appears to have been some non-compliance with recommendations over the past decade, despite evidence suggesting that compliance with management recommendations is associated with superior outcomes [32,33]. Particularly with regard to the management of HF, the attitudes of primary care practitioners have revealed substantial disparities. HF appears to be a challenging clinical condition that continues to be a concern for healthcare professionals [11,34]. This necessitates interventions in these groups to improve the implementation of guidelines when necessary. Compared to other maladies, the current public perception of heart failure and civic efforts appear insufficient for healthcare authorities, medical professionals, and other stakeholders to develop appropriate measures.

Together, our findings and those of the SHAPE survey should encourage HF awareness activities among the general public and beyond [13]. The Heart Failure Association at the European Society of Cardiology is sincerely trying to increase public and important stakeholder awareness of this dangerous and crippling condition through the Heart Failure Awareness Day initiative. The initiative has grown quantitatively and qualitatively over the past three years. The current report and studies such as SHAPE provide us with the knowledge necessary for future activity planning to maximize results and benefits. In an ideal world, activities should not be limited to one-day or week-long events like the European Heart Failure Awareness Day but should instead expand into a continuous endeavor for the general public, healthcare groups, and policymakers. Simultaneously, changes in national healthcare policies should be pursued to entrust healthcare institutions with continuous promotion and iterative campaigns about healthy lifestyles and preventive activities to reduce disease-associated costs and disability. An infrastructure capable of deployment across a substantial portion of Europe appears to be in place [35], paving the way for a coordinated and standardized effort.

Public education campaigns about heart failure in Saudi Arabia can have a major impact on healthcare policy and intervention efforts aimed at reducing the prevalence of heart failure in the population. This is because an informed public is more likely to take preventative measures and seek medical care for heart failure when it is still in its early stages, thereby increasing the likelihood of a positive outcome. Heart failure awareness campaigns can also call attention to the need for healthcare that is both universally available and reasonably priced. In addition, if people are made more aware of the disease, they will be better able to take an active role in its management by learning about its symptoms, available treatments, and self-care techniques.

This study has limitations. The sample size was relatively small, which could have limited the power of the study to detect significant associations. The use of the convenience sampling technique is not free from criticism as it might affect the generalizability of our findings. The online study design also might affect the generalizability of our study findings. However, the percentage of Saudi Arabians who use social media, according to the most recent data from 2023, is close to 79.3%. In addition, unfortunately, we did not estimate the number of individuals who received an invitation to participate in this online survey; therefore, we were not able to present the response rate, which might lead to non-response bias. Therefore, our findings should be interpreted carefully.

## Conclusions

In Saudi Arabia, there was little general public awareness about HF. We advise spreading awareness about HF through an educational campaign launched by medical professionals and shared on various social media platforms. To reduce disease-related expenditures and disability, national healthcare policies should be changed to offer healthcare institutions ongoing promotion and iterative campaigns about healthy lifestyles and preventative behaviors. To raise HF awareness, more attention and instruction are required.

## Appendices

Appendix 1

Table 5 presents the questions used to assess knowledge regarding CV disease and HF.

**Table 5:** Questions used to assess knowledge regarding CV disease and HF HF: heart failure, CV: cardiovascular

Awareness regarding cardiovascular and cerebrovascular disorders
Chest heaviness that occurs during exertion and disappears with rest
Facial paralysis, double vision, and sudden unilateral weakness in the arm
Breathlessness, tiredness, and swollen ankles
Awareness regarding HF, its etiology, and severity
Words that best describe heart failure
Perceived severity if you have the following symptoms: breathlessness, tiredness, or swollen ankles
How soon will you go to the hospital if you feel breathlessness, tiredness, or swollen ankles?
Agree that heart failure is a normal aging process
Awareness regarding the risk of HF
Correct lifetime risk of developing heart failure
Post-discharge one-year mortality from acute heart failure
Readmission rate within one year after discharge from heart failure

 Table 6 shows the education and awareness of male and female respondents regarding HF and other CV diseases.

**Table 6:** Education and awareness regarding HF and other CV diseases HF: heart failure, QoL: quality of life, MI: myocardial infarction, GI: gastrointestinal

Variable	Female	Male	P-value
	N = 672	N = 452	
Chest heaviness that occurs during exertion and disappears with rest			0.413
Angina or MI	104 (36%)	340 (40.7%)	
GI disorders	17 (5.88%)	40 (4.79%)	
I do not know	53 (18.3%)	120 (14.4%)	
Lung disorders	40 (13.8%)	114 (13.7%)	
Other heart diseases	75 (26%)	221 (26.5%)	
Facial paralysis, double vision, and sudden unilateral weakness in the arm			0.001
Angina or MI	38 (13.1%)	196 (23.5%)	
I do not know	63 (21.8%)	149 (17.8%)	
Other heart diseases	24 (8.30%)	72 (8.62%)	
Parkinson's disease, epilepsy, and other brain diseases	64 (22.1%)	129 (15.4%)	
Stroke	100 (34.6%)	289 (34.6%)	
Breathlessness, tiredness, and swollen ankles			0.479
Angina or MI	33 (11.4%)	81 (9.70%)	
Heart diseases in general	76 (26.3%)	189 (22.6%)	
Heart failure*	83 (28.7%)	281 (33.7%)	
I do not know	53 (18.3%)	153 (18.3%)	
Lung disorders	44 (15.2%)	131 (15.7%)	
Ever heard of heart failure			0.487
No	166 (57.4%)	458 (54.9%)	
Yes	123 (42.6%)	377 (45.1%)	
Words that best describe heart failure			0.001
Abnormality of heart rhythm	27 (9.34%)	45 (5.39%)	
I do not know	49 (17%)	114 (13.7%)	
Lack of oxygen and blood supply to the heart due to myocardial infarction	64 (22.1%)	159 (19%)	
The heart cannot pump enough blood around the body	118 (40.8%)	454 (54.4%)	
Weakness of the heart by aging	31 (10.7%)	63 (7.54%)	
Perceived severity if you have the following symptoms: breathlessness, tiredness, or swollen ankles			0.119
I do not know	43 (14.9%)	91 (10.9%)	
Minor illness	45 (15.6%)	108 (12.9%)	
Serious illness	85 (29.4%)	290 (34.7%)	
Slightly serious illness	116 (40.1%)	346 (41.4%)	
How soon will you go to the hospital if you feel breathlessness, tiredness, or swollen ankles?			0.035
Never	12 (4.15%)	22 (2.63%)	
One to two days	171 (59.2%)	541 (64.8%)	
Within 1-3 weeks	37 (12.8%)	61 (7.31%)	
Within one month	12 (4.15%)	34 (4.07%)	
Within one week	57 (19.7%)	177 (21.2%)	
History/family history of heart disease			0.740
No	196 (67.8%)	577 (69.1%)	
Yes	93 (32.2%)	258 (30.9%)	
Diagnosis			0.457
Abnormality of heart rhythm	21 (9.91%)	46 (7.90%)	
Angina or MI	31 (14.6%)	66 (11.3%)	
Heart failure	32 (15.1%)	78 (13.4%)	
I do not know	95 (44.8%)	295 (50.7%)	
Valvular heart disease	33 (15.6%)	97 (16.7%)	
Agree that heart failure is a normal aging process			0.433
No	138 (47.8%)	423 (50.7%)	
Yes	151 (52.2%)	412 (49.3%)	
Causes of heart failure			0.722
Hypertension	239 (82.7%)	700 (83.8%)	
Lung disorders	50 (17.3%)	135 (16.2%)	
Correct lifetime risk of developing heart failure			0.307
2 in 100 people	70 (24.2%)	212 (25.4%)	
5 in 100 people	91 (31.5%)	290 (34.7%)	
10 in 100 people	79 (27.3%)	227 (27.2%)	
20 in 100 people	49 (17%)	106 (12.7%)	
Highest mortality within five years after diagnosis			0.038
Heart failure	98 (33.9%)	338 (40.5%)	
Myocardial infarction	91 (31.5%)	247 (29.6%)	
Prostate or breast cancer	22 (7.61%)	82 (9.82%)	
Stroke	78 (27%)	168 (20.1%)	
Worried that one of your friends, colleagues, or neighbors who suffers from heart failure might suddenly die			0.506
I do not know	56 (19.4%)	157 (18.8%)	
No	38 (13.1%)	90 (10.8%)	
Yes	195 (67.5%)	588 (70.4%)	
Post-discharge one-year mortality from acute HF			0.002
2 in 100 people	85 (29.4%)	184 (22%)	
5 in 100 people	55 (19%)	197 (23.6%)	
10 in 100 people	61 (21.1%)	124 (14.9%)	
20 in 100 people	17 (5.88%)	65 (7.78%)	
I do not know	71 (24.6%)	265 (31.7%)	
Readmission rate within one year after discharge from HF			0.003
2 in 100 people	64 (22.1%)	152 (18.2%)	
5 in 100 people	71 (24.6%)	193 (23.1%)	
10 in 100 people	61 (21.1%)	120 (14.4%)	
20 in 100 people	16 (5.54%)	67 (8.02%)	
I do not know	77 (26.6%)	303 (36.3%)	
Average healthcare costs per admission from acute heart failure			0.400
<10,000	55 (19%)	134 (16%)	
>80,000	52 (18%)	154 (18.4%)	
10,000-20,000	65 (22.5%)	196 (23.5%)	
30,000-50,000	80 (27.7%)	210 (25.1%)	
60,000-80,000	37 (12.8%)	141 (16.9%)	
Disease with the greatest impact on the quality of life			0.107
Arthritis	24 (8.30%)	49 (5.87%)	
Diabetes	61 (21.1%)	170 (20.4%)	
Heart failure	154 (53.3%)	504 (60.4%)	
Hypertension	50 (17.3%)	112 (13.4%)	
Preferred treatments if you were a heart failure patient			0.458
I can not decide	59 (20.4%)	200 (24%)	
Treatment that could improve the quality of life	193 (66.8%)	529 (63.4%)	
Treatment that prolongs survival	37 (12.8%)	106 (12.7%)	
Current heart failure medications could reduce death from HF			0.101
No	36 (12.5%)	73 (8.74%)	
I do not know	93 (32.2%)	310 (37.1%)	
Yes	160 (55.4%)	452 (54.1%)	
Current heart failure medications could improve the QoL in patients with HF			0.667
No	22 (7.61%)	51 (6.11%)	
I do not know	87 (30.1%)	253 (30.3%)	
Yes	180 (62.3%)	531 (63.6%)	
Current heart failure medications could prevent the occurrence of HF			0.627
No	72 (24.9%)	208 (24.9%)	
I do not know	107 (37%)	333 (39.9%)	
Yes	110 (38.1%)	294 (35.2%)	
Heart failure patients should live quietly and reduce all physical activity			0.410
No	57 (19.7%)	196 (23.5%)	
I do not know	65 (22.5%)	184 (22%)	
Yes	167 (57.8%)	455 (54.5%)	
Awareness regarding CV diseases (mean (SD))	0.99 (0.92)	1.09 (0.98)	0.130
Awareness regarding heart failure (mean (SD))	1.77 (1.06)	2.05 (1.11)	<0.001
Overall awareness (mean (SD))	3.05 (1.59)	3.42 (1.79)	0.001
